# Protective and risk factors in daily life associated with cognitive decline of older adults

**DOI:** 10.3389/fnagi.2025.1496677

**Published:** 2025-02-26

**Authors:** Fang Tong, Hao Yang, Haidong Yu, Le-wen Sui, Jing-yuan Yao, Chen-lei Shi, Qiao-yuan Yao, Mei-fang Shi, Cheng-lang Qian, Gang Li, Chao Zhao, Hui-jing Wang

**Affiliations:** ^1^Institute of Wound Prevention and Treatment, Shanghai University of Medicine & Health Sciences, Shanghai, China; ^2^Department of Physiology, School of Fundamental Medicine, Shanghai University of Medicine & Health Sciences, Shanghai, China; ^3^Department of Neurology, Zhoupu Hospital Affiliated to Shanghai University of Medicine & Health Sciences, Shanghai, China; ^4^National Clinical Research Center for Aging and Medicine, Huashan Hospital, Shanghai Medical College, Fudan University, Shanghai, China; ^5^Department of General Medicine, Youyi Road Community Health Service Centre for Baoshan District, Shanghai, China; ^6^Department of General Medicine, Huadong Hospital, Fudan University, Shanghai, China; ^7^Department of Pharmacology, School of Fundamental Medicine, Shanghai University of Medicine & Health Sciences, Shanghai, China

**Keywords:** protective factors, risk factors, cognitive decline, older adults, cognitive function

## Abstract

**Background:**

Cognitive decline is a chronic condition which is characterized by a loss of the ability to remember, learn, and pay attention to complex tasks. Many older people are now suffering from cognitive decline, which decreases life quality and leads to disability. This study aimed to identify the risk and protective factors for cognitive decline of the older people from daily life and establish a predictive model using logistic regression.

**Methods:**

We investigated 3,790 older people with health examination and questionnaires which included information associated with physical condition, lifestyle factors, and cognitive status. Single-factor comparison, principal component analysis with a Manova-Wilk test, multiple linear regression, and logistic regression were performed to filter the risk and protective factors regarding cognitive decline of older individuals. Then a predictive model using logistic regression was established based on the most significant protective and risk factors.

**Results:**

We found a significant separation along the coordinate axis between people with normal and declined cognition by principal component analysis, as confirmed by the Manover-Wilk test. Single-factor comparison, multiple linear regression and logistic regression implied that gender, age, hypertension level, height, dietary habit, physical-exercise duration, physical-exercise history, and smoking history could be closely linked with cognitive decline. We also observed significant differences in height, physical exercise duration, physical-exercise years, and smoking years between the male and female of the participants. ROCs of the predictive model by logistic regression were plotted, with AUC values of 0.683 and 0.682, respectively, for the training and testing sets. Although an effective predictive model is thought to have AUC over 0.7, we still believe that the present model is acceptable because the value is close to the threshold.

**Conclusion:**

The protective factors of cognitive decline for older people were male gender, height, keeping moderate exercising, and nicotine stimulation, and the risk factors included age, female gender, vegetarianism and hypertension. Except for the genetic factor, differences in lifestyle, such as smoking and exercise habits, may contribute to the observed differences in cognitive function between genders. The significant results could be utilized in the practice for the early intervention of cognitive decline in aged people.

## Introduction

Cognitive decline is a chronic condition which is characterized by a loss of the ability to remember, learn, and pay attention to complex tasks, incomparable with the educational level (McKhann et al., 2011; Dominguez et al., 2021). The clinical manifestation of cognitive decline is minor and difficult to observe in the early stage, and will eventually proceed to dementia without medical intervention. Globally, approximately 47 million people have been diagnosed with dementia and this number is increasing by 8 million annually (Dominguez et al., 2021). The dementia population is expected to be close to 140 million in 2050 (Prince et al., 2016). Aging is proved to be positively associated with cognitive decline. For older people, cognition determines life expectancy, as well as life quality, including independent living, effective communication, finance management, drug administration and safe driving. Age-related cognitive decline is a serious issue which human beings must confront in this century. Nowadays, the life expectancy of most people is over 60 (Beard et al., 2016), and children alive are extremely optimistic that they could live beyond 100 years (Vaupel, 2010). Many older people are suffering from cognitive decline, which is decreasing life quality and may lead to disability (Prince et al., 2015). Alzheimer’s disease (AD) and vascular dementia are the top causes of dementia in older people (Scheltens et al., 2021; Murman, 2015). AD is a neurodegenerative disorder with unknown etiology, and the number of AD patients will exceed 13.8 million by 2050 (Xu et al., 2023). Unfortunately, no effective method has been established to treat dementia. Thus, risk and protective factors of cognitive decline should be disclosed to make appropriate prevention.

Cognitive performance can be assessed using various psychological scales, such as the Mini-Mental State Examination (MMSE), Montreal Cognitive Assessment (MoCA), Rowland Universal Dementia Assessment Scale (RUDAS), and Cognitive Impairment Scale (PAS). Among these scales, MMSE is widely used in clinical and research settings due to its brevity and usefulness. It is a brief questionnaire consisting of 30 points that can sensitively test cognitive decline (Arevalo-Rodriguez et al., 2021). MMSE is utilized to gauge the severity and progression of cognitive decline, as well as to monitor the evolution of cognitive changes in an individual over time (Shim et al., 2017). The examination is made up of 8 sections which are orientation to time, orientation to place, registration, attention and calculation, recall, language, repetition, and complex commands. People with normal cognition have an MMSE score over 24, and scores below this indicate cognitive decline of different degrees: severe (0–9 points), moderate (10–18 points), and mild (19–23 points) (Crum et al., 1993).

Risk and protective factors of cognitive decline are complicated and multifaceted, including aging, living habits, nutrition, educational level, vascular pathology, sleep-disordered breathing, Apolipoprotein E (APOE) genotype, lack of Vitamin D, and earlier severe diseases (Yaffe et al., 2011; Iwashyna et al., 2010). Many research, nowadays, are looking for genes related to cognitive decline to diagnose cognitive decline at the early stage to make timely intervention before the situation deteriorates (Xu et al., 2023). Advanced age is an independent cause for cognitive decline, and other risk factors are biological, socioeconomic, and environmental, which can be intervened to reduce the incidence rate (Dominguez et al., 2021). Our study team would like to find the methods to decrease the risk of cognitive decline based on healthy lifestyles. This study conducted in the Youyi community of Shanghai investigated 3,790 older people with health examination and questionnaires which included information associated with physical condition, lifestyle factors, and cognitive status. We aimed to identify the risk and protective factors for cognitive decline from daily life and establish a predictive model using logistic regression based on the results of the questionnaire. The significant results could be utilized in practice to support early interventions for cognitive decline in older adults.

## Methods

### Participants and study design

**Figure 1:**
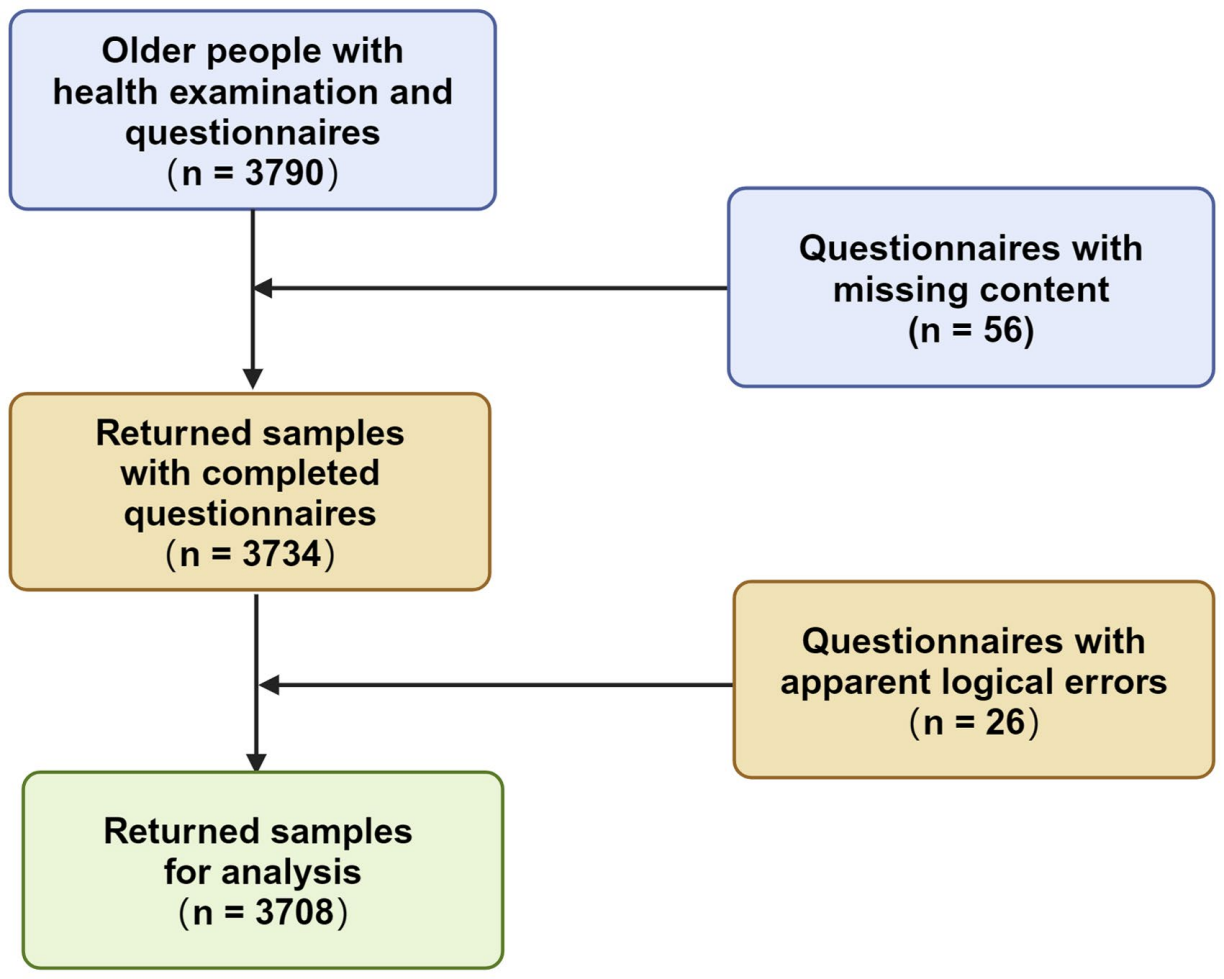
Inclusion procedure of the study (Created with BioRender.com).

The study included participants who took part in a health examination organized by a community hospital in the Baoshan district of Shanghai. The data were collected from senior citizens residing in the Youyi Community in November 2020, during a period of strict COVID-19 policy enforcement in Shanghai. This ensured that the impact of virus exposure could be excluded. Participants provided informed consent prior to the health examination. All the participants declared to have no cancer or other life-threatening diseases. The survey, conducted by experienced clinical doctors from the hospital, comprised three parts: general physical condition (including blood pressure, respiratory rate, height, weight, BMI), lifestyle habits (diet, physical exercise frequency and duration, years of exercise, smoking habits, daily cigarette consumption, years of smoking, alcohol consumption, years of drinking), and cognitive status (assessed using the MMSE). 3,790 participants were recruited, and the project duration was approximately half a month. They were required to answer a questionnaire before the clinical parameters were measured. The questionnaire of the present study has never been published before and could be attached in the Supplementary material 1. Questionnaires with missing content or apparent logical errors were excluded from the analysis. A total of 3,708 samples were returned, yielding an effective response rate of 97.83%. Figure 1 presented a flowchart illustrating the inclusion procedure. The study received approval from the Ethic Review Board of Shanghai University of Medicine and Health Sciences and Shanghai Medical College of Fudan University.

### Definition, classification, and assignment

Cognitive status was defined and classified based on the MMSE score. Participants with an MMSE score above 24 were assigned to the normal cognition group (assigned as 0), while those with a score between 0 and 23 were categorized as having cognitive decline (assigned as 1). Gender was assigned as 0 for females and 1 for males. Blood pressure (BP) was classified according to the American Heart Association’s BP category standards and assigned different values from 0 to 4. Self-assessment of health condition, dietary habits, physical exercise frequency, smoking addiction level, and alcohol addiction level were also classified and assigned specific values. More information on the classification and assignment of variables can be found in Supplementary material 2.

### Statistical analysis

Characteristics were statistically described as mean ± standard error mean (SEM). Basic statistical analysis and graph generation were performed using GraphPad Prism 10.0 software (GraphPad Software, San Diego, CA, USA). Multiple linear regression with stepwise regression was performed by IBM SPSS 20. Principal Component Analysis (PCA) with Manova-Wilk test, logistic regression, and nomograph were performed based on R Studio (software version 4.2.2). *p* value thresholds are set to 0.1 (weak evidence), 0.05 (moderate evidence), 0.01 (strong evidence), 0.001 (very strong evidence) and 0.0001 (extremely strong evidence). For the predictive model, samples were randomly assigned to a training set (70%) and a test set (30%). The model was built based on the training set and the accuracy was checked on the test set. We share all the codes associated with analysis on R studio in the Supplementary material 3.

## Results

### Sample distribution and variables

**Figure 2:**
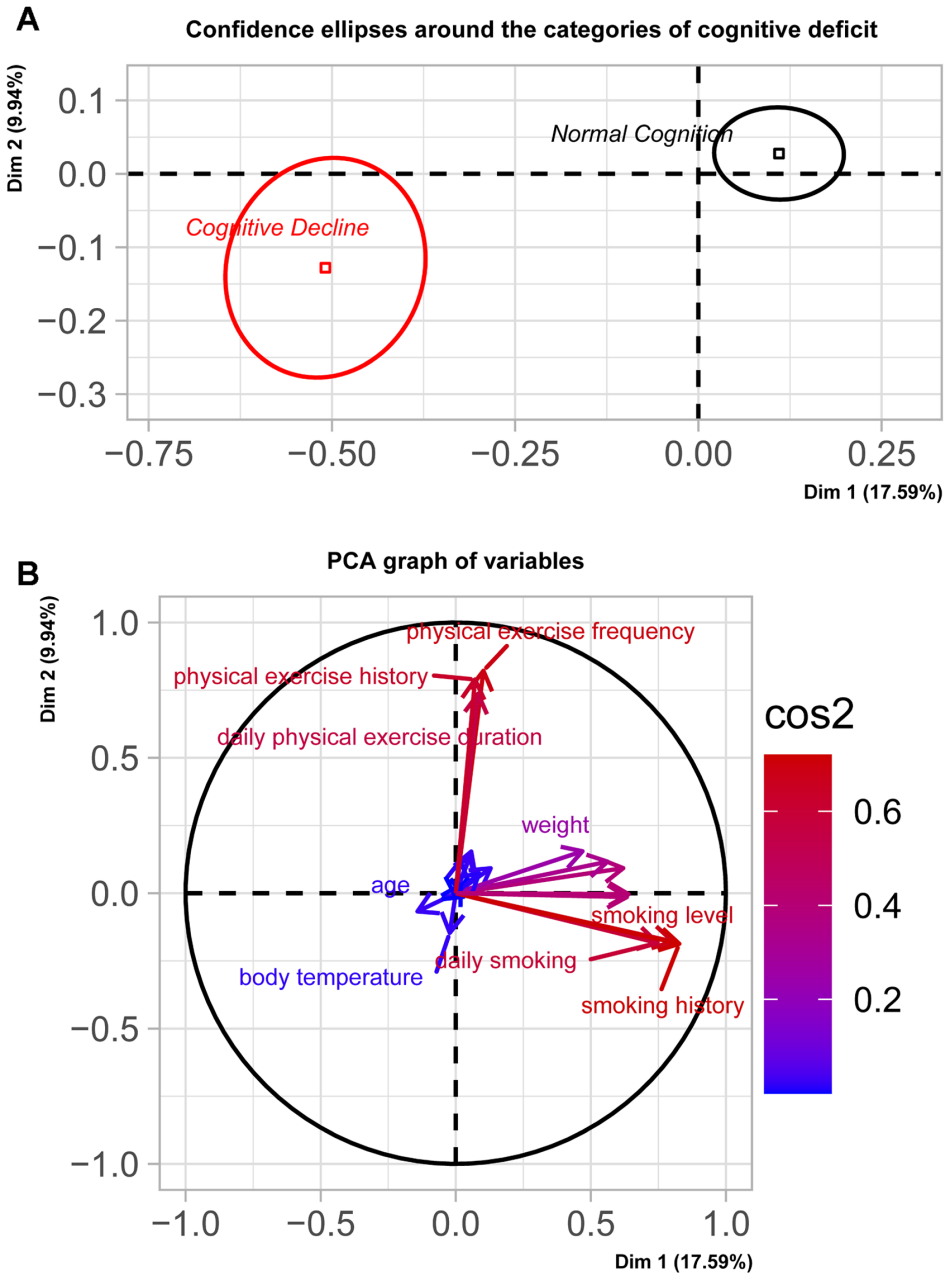
Sample distribution and principle components. **(A)** illustrated the distribution of samples from groups with normal cognition and cognitive decline. A significant separation along the coordinate axis was observed, as confirmed by the Manover-Wilk test (*p* < 0.0001). **(B)** demonstrated the principal components of the two dimensions. Dimension 1 contrasts individuals with a strongly positive coordinate on the axis (right side of the graph) against those with a strongly negative coordinate (left side of the graph). Dimension 2 contrasted individuals with a strongly positive coordinate on the axis (top of the graph) against those with a strongly negative coordinate (bottom of the graph).

Figure 2A illustrated the distribution of samples from two groups: those with normal cognition and those experiencing cognitive decline. A significant separation along the coordinate axis was observed, as confirmed by Manover-Wilk test (*p* < 0.0001). Figure 2B presented the principal components of the two dimensions. Dimension 1 contrasted individuals with a strongly positive coordinate on the axis (right side of the graph) against those with a strongly negative coordinate (left side of the graph). Variables such as years of smoking, smoking addiction, daily cigarette consumption, years of drinking, drinking addiction, gender, height, weight, frequency of physical exercise, and self-assessment of health condition significantly influence the sample position on the positive coordinate axis (indicating normal cognition). Conversely, variables like body temperature and age significantly influence the sample position on the negative coordinate axis (indicating cognitive decline). Dimension 2 contrasted individuals with a strongly positive coordinate on the axis (top of the graph) against those with a strongly negative coordinate (bottom of the graph). Variables such as frequency of physical exercise, years of physical exercise, duration of physical exercise, hypertension level, diastolic pressure, systolic pressure, pulse rate, and BMI significantly influence the sample position on the positive coordinate axis (indicating normal cognition). Again, variables like body temperature and age significantly influenced the sample position on the negative coordinate axis (indicating cognitive decline). For more details on the correlation values of these variables, please refer to Supplementary material 4.

Figure 2A illustrated the distribution of samples from groups with normal cognition and cognitive decline. A significant separation along the coordinate axis was observed, as confirmed by the Manover-Wilk test (*p* < 0.0001). Figure 2B demonstrated the principal components of the two dimensions. Dimension 1 contrasts individuals with a strongly positive coordinate on the axis (right side of the graph) against those with a strongly negative coordinate (left side of the graph). Dimension 2 contrasted individuals with a strongly positive coordinate on the axis (top of the graph) against those with a strongly negative coordinate (bottom of the graph).

### Characteristics of samples

Table 1 presented the baseline characteristics of all samples, categorized by normal cognition and cognitive decline. Of the 3,708 individuals, 3,048 exhibited normal cognition, while 660 showed signs of cognitive decline. The group with cognitive decline had a higher proportion of females (*p* < 0.0001) and were generally older (*p* < 0.0001) than those with normal cognition. This group also had a lower proportion of individuals with normal and elevated blood pressure, but a higher proportion with high blood pressure (*p* < 0.0001). Specifically, the systolic pressure demonstrated a significant increase in group of cognitive decline (*p* < 0.01), but the diastolic pressures did not. Individuals with normal cognition had higher pulse rates (*p* < 0.05), as well as greater height and weight (*p* < 0.0001 for both) than those with cognitive decline. Dietary habits between the two groups showed weakly significant differences (*p* < 0.1), with the cognitive decline group having a higher proportion of individuals consuming ‘vegetables only’ and ‘more vegetables than meat.’ In terms of physical exercise, the cognitive decline group had a higher proportion of individuals who ‘never exercise’ (*p* < 0.01) and a longer average duration of physical exercise, although the latter was only weakly significant (*p* < 0.1). The normal cognition group, on the other hand, had more years of physical exercise (*p* < 0.05). Regarding smoking habits, 92.09% of the normal cognition group declared ‘never smoke,’ compared to 96.97% in the cognitive decline group (*p* < 0.0001). The normal cognition group smoked approximately 1.007 cigarettes per day, which was higher than the 0.3985 per day in the cognitive decline group (*p* < 0.0001). People from the group with normal cognition also had around 2 more years of smoking (*p* < 0.0001) than the other. In terms of alcohol addiction, 93.73% of the normal cognition group reported never having smoked, compared to 96.97% in the cognitive decline (*p* = 0.0013). The ‘drinking years’ for the normal cognition group was approximately 2.83, compared to 1.455 for the cognitive decline group (*p* < 0.01). No significant differences were observed in body temperature, pulse rate, self-assessment of health, respiratory rate, and BMI between the two groups.

**Table 1:** Comparison of the characteristics.

Characteristics	Total (*N* = 3,708)	Normal cognition (*n* = 3,048)	Cognitive decline (*n* = 660)	*p* value
Gender				
Male	1,466 (39.54%)	1,285 (42.16%)	181 (27.42%)	<0.0001^****^
Female	2,242 (60.46%)	1,763 (57.84%)	479 (72.58%)	
Age (years)	72.05 (0.09199)	71.52 (0.09533)	74.51 (0.2496)	<0.0001^****^
Blood pressure (mmHg)				
Diastolic pressure	76.51 (0.1041)	76.56 (0.1139)	76.28 (0.2553)	0.3334
Systolic pressure	129.5 (0.1595)	129.3 (0.1766)	130.2 (0.3706)	0.0011^**^
Hypertension level^a^				
No hypertension	197 (5.31%)	148 (4.86%)	31 (4.70%)	<0.0001^****^
Elevated	1,366 (36.84%)	1,167 (38.29%)	199 (30.15%)	
High blood pressure stage 1	1,777 (47.92%)	1,408 (46.19%)	369 (55.91%)	
High blood pressure stage 2	382 (10.30%)	323 (10.60%)	59 (8.94%)	
High blood pressure crisis	4 (0.11%)	2 (0.066%)	2 (0.30%)	
Body temperature (°C)	36.55 (0.003612)	36.55 (0.003939)	36.55 (0.009002)	0.7808
Pulse rate (/min)	73.48 (0.06783)	73.52 (0.07385)	73.33 (0.17)	0.0265^*^
Self-assessment of health^a^				
Dissatisfactory	1 (0.03%)	1 (0.03%)	0 (0%)	0.8612
Not quite satisfactory	9 (0.24%)	8 (0.26%)	1 (0.15%)	
Basically satisfactory	3,489 (94.09%)	2,871 (94.19%)	618 (93.64%)	
Satisfactory	204 (5.50%)	165 (5.41%)	39 (5.91%)	
Respiratory rate (/min)	18.12 (0.0205)	18.12 (0.02258)	18.12 (0.04898)	0.9004
Height (cm)	161.6 (0.1354)	162.1 (0.1492)	159.0 (0.3035)	<0.0001^****^
Weight (kg)	63.48 (0.1641)	63.90 (0.182)	61.55 (0.3696)	<0.0001^****^
BMI	24.35 (0.07598)	24.36 (0.08822)	24.32 (0.1274)	0.9135
Dietary habit^a^				
Vegetable only	42 (1.13%)	29 (0.95%)	13 (1.97%)	0.054^#^
More vegetables than meat	6 (0.16%)	4 (0.13%)	2 (0.30%)	
Balanced diet	3,635 (98.03%)	2,991 (98.13%)	644 (97.58%)	
More meat than vegetables	1 (0.03%)	1 (0.03%)	0 (0%)	
Meat only	24 (0.65%)	23 (0.75%)	1 (0.15%)	
Physical-exercise frequency				
Never do exercise	1,091 (29.42%)	859 (28.18%)	232 (35.15%)	0.0044^**^
Do exercise occasionally	69 (1.86%)	59 (1.94%)	10 (1.52%)	
More than once a week	38 (1.02%)	33 (1.08%)	5 (0.76%)	
Every day	2,510 (67.69%)	2097 (68.80%)	413 (62.58%)	
Physical-exercise duration (min)	34.62 (0.6196)	34.60 (0.6203)	34.72 (1.979)	0.0685^#^
Physical-exercise years	7.392 (0.1081)	7.484 (0.1182)	6.968 (0.2653)	0.013^*^
Smoking addiction^a^				
Never smoke	3,447 (92.96%)	2,807 (92.09%)	640 (96.97%)	<0.0001^****^
Used to smoke, not smoke now	69 (1.86%)	62 (2.03%)	7 (1.06%)	
Smoke now, but not every day	7 (0.19%)	5 (0.16%)	2 (0.30%)	
Smoke everyday	185 (4.99%)	174 (5.71%)	11 (1.67%)	
Daily-cigarette number	0.8986 (0.06543)	1.007 (0.07496)	0.3985 (0.1219)	<0.0001^****^
Smoking years	2.934 (0.1851)	3.332 (0.2164)	1.092 (0.2766)	<0.0001^****^
Alcohol addiction^a^				
Never drink	3,497 (94.31)	2,857 (93.73)	640 (96.97)	0.0013^**^
Drink occasionally	63 (1.70)	60 (1.97)	3 (0.45)	
Always drink but not every day	21 (0.57)	18 (0.59)	3 (0.45)	
Drink everyday	127 (3.43)	113 (3.71)	14 (2.12)	
Drinking years	2.585 (0.1809)	2.830 (0.2073)	1.455 (0.3385)	0.0024^**^

a Chi-square test is used for the comparison of the classification variables. ^#^*p* < 0.1, *p < 0.05, **p < 0.01, ***p < 0.001, ****p < 0.0001.

### Results from multiple linear regression and logistic regression

A multiple linear regression with stepwise regression was primarily performed to weigh the correlation between MMSE value and the multiple continuous variables of clinical measurements, such as age, blood pressure, body temperature, pulse rate, respiratory rate, height, and weight, which can be conveniently measured in the clinical practice. Seeing the close association of BMI with height and weight, BMI was excluded in the linear regression analysis. As is demonstrated in Table 2, the characteristics of age, body temperature, height, and systolic pressure were significantly related to MMSE score. Specifically, age was negatively associated with MMSE score (*p* < 0.0001, coefficient = −1.75, 95%CI: −0.196–-0.154), while body temperature (*p* < 0.05, coefficient = 0.129, 95%CI: 0.014–1.096), height (*p* < 0.0001, coefficient = 0.730, 95%CI: 0.055–0.084) and systolic pressure (*p* < 0.0001, coefficient = 0.128, 95%CI: 0.001–0.023) were positively related to MMSE score. A multiple linear model was also reported here: MMSE = 5.690–1.75 × age + 0.73 × height + 0.128 × systolic pressure + 0.129 × body temperature (adjusted R^2^ = 0.094, S.E = 3.692, DW test = 1.905).

**Table 2:** Results from multiple linear regression with stepwise regression.

Characteristics	Coefficient	S.E	*p* value	Zero-order correlation	95% CI
Age	−1.75	0.011	<0.0001^****^	−0.266	(−0.196, −0.154)
Height	0.730	0.007	<0.0001^****^	0.171	(0.055, 0.084)
Systolic pressure	0.128	0.005	0.026^*^	0.045	(0.001, 0.023)
Body temperature	0.129	0.276	0.044^*^	0.034	(0.014, 1.096)

^#^*p* < 0.1, *p < 0.05, **p < 0.01, ***p < 0.001, ****p < 0.0001.

**Figure 3:**
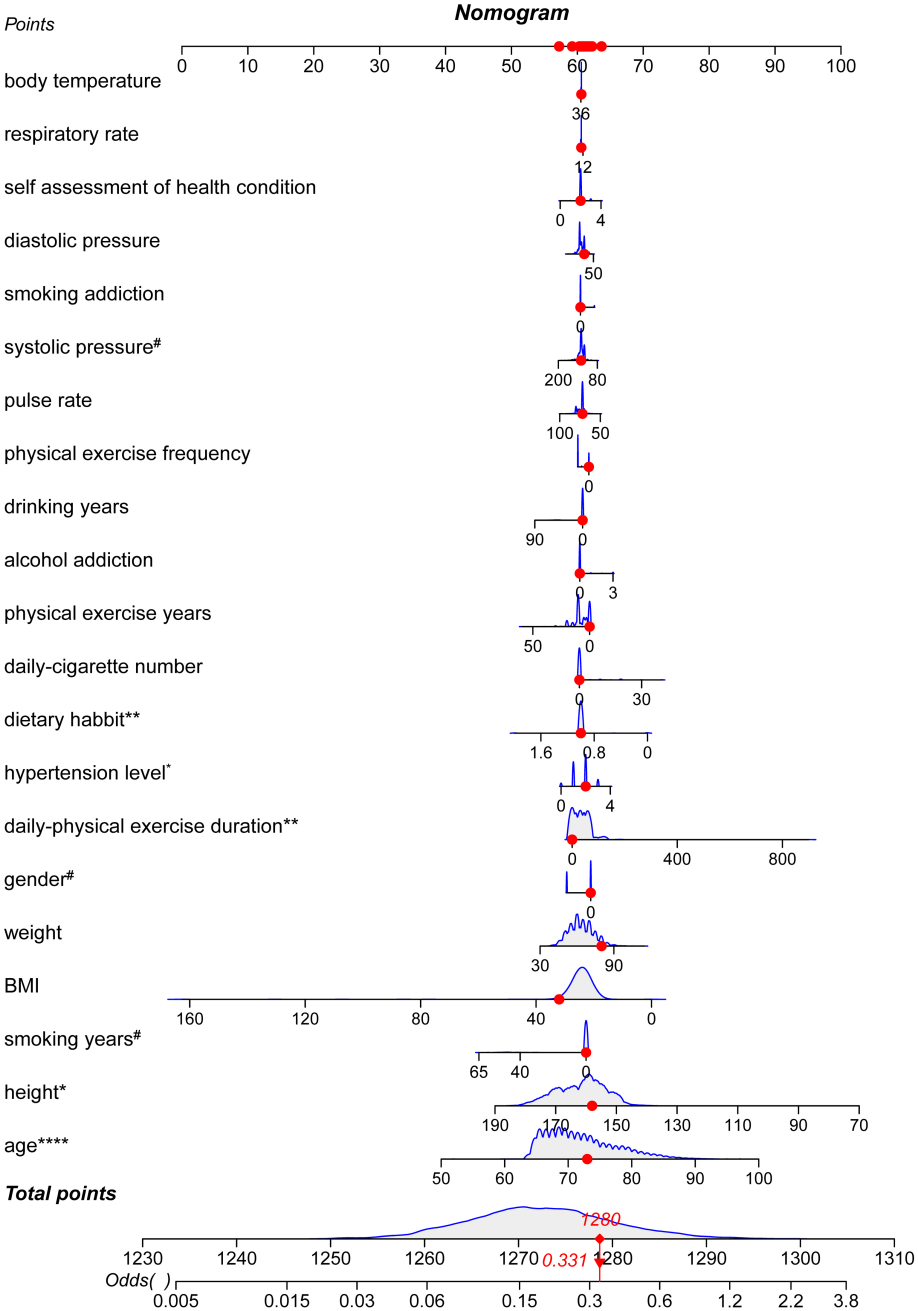
Nomograph of the logistic regression. The nomograph demonstrated the normalized weight of each parameter by a score ranging from 0 to 100. The gray areas with blue outlines demonstrated the sample distribution in each parameter. A sample was randomly chosen, and the red points represented the position as well as the score for a factor. This sample got 1280 points of 1310 and the odds value is 0.331. (^#^*p* < 0.1, *p < 0.05, **p < 0.01, ****p < 0.0001).

Table 3 presented the risk and protective factors of cognitive decline determined resulted from a univariate logistic regression model. Significant positive-related factors for cognitive decline included age (*p* < 0.0001, OR = 1.9353, 95%CI: 1.7015–2.2012), hypertension level (*p* < 0.05, OR = 1.3031, 95%CI: 1.0536–1.6117), and physical-exercise duration (*p* < 0.01, OR = 1.2563, 95%CI: 1.0695–1.4757). Significant negative-related factors for cognitive decline were male gender (*p* < 0.1, OR = 0.7327, 95%CI: 0.525–1.023), systolic pressure (*p* < 0.1, OR = 0.8966, 95%CI: 0.7892–1.0187), dietary habit (*p* < 0.05, OR = 0.1921, 95%CI: 0.0431–0.8562), physical-exercise years (*p* < 0.1, OR = 0.8303, 95%CI: 0.6676–1.0326), and smoking years (*p* < 0.1, OR = 0.1433, 95%CI: 0.0197–1.0437). These results are visualized in a nomograph (Figure 3).

**Table 3:** Results from logistic regression.

Characteristics	Coefficient	S.E	*p* value	Odds ratio	95% CI
Gender	−0.3111	0.1702	0.0675^#^	0.7327	(0.525, 1.023)
Age	0.0943	0.0094	<0.0001^****^	1.9353	(1.7015, 2.2012)
Hypertension level	0.2647	0.1084	0.0146^*^	1.3031	(1.0536, 1.6117)
Diastolic pressure	−0.0015	0.0088	0.8667	0.9883	(0.8616, 1.1337)
Systolic pressure	−0.0136	0.0081	0.0938^#^	0.8966	(0.7892, 1.0187)
Body temperature	−0.0718	0.2458	0.7701	0.9787	(0.8467, 1.1309)
Pulse rate	−0.0115	0.0129	0.3706	0.9550	(0.8634, 1.0563)
Self-assessment of health	0.0874	0.1840	0.6348	1.4185	(0.3353, 6.0016)
Respiratory rate	−0.0108	0.0413	0.8783	0.86465	(0.3328, 2.318)
Height	−0.0400	0.0169	0.0181^*^	0.6187	(0.4156, 0.9211)
Weight	0.0168	0.0155	0.2794	1.2647	(0.8264, 1.9354)
BMI	−0.0376	0.0328	0.2523	0.8637	(0.6721, 1.11)
Dietary habit	−0.8248	0.3812	0.0305^*^	0.1921	(0.0431, 0.8562)
Physical exercise					
Physical-exercise frequency	−0.0705	0.0525	0.1792	0.8094	(0.6721, 1.11)
Daily-physical-exercise duration	0.0038	0.0014	0.0055^**^	1.2563	(1.0695, 1.4757)
Physical-exercise years	−0.0186	0.0111	0.0946^#^	0.8303	(0.6676, 1.0326)
Smoking					
Smoking addiction	0.2155	0.2161	0.3188	1.9086	(0.5355, 6.8022)
Daily-cigarette number	0.0398	0.0280	0.1556	4.9055	(0.5463, 44.047)
Smoking years	−0.0324	0.0169	0.0551^#^	0.1433	(0.0197, 1.0437)
Drinking					
Alcohol addiction	0.2031	0.2137	0.3419	1.8389	(0.5235, 6.460)
Drinking years	−0.0071	0.0115	0.5392	0.5643	(0.09083, 3.505)

^#^*p* < 0.1, *p < 0.05, **p < 0.01, ***p < 0.001, ****p < 0.0001.

The nomograph demonstrated the normalized weight of each parameter by a score ranging from 0 to 100. The gray areas with blue outlines demonstrated the sample distribution in each parameter. A sample was randomly chosen, and the red points represented the position as well as the score for a factor. This sample got 1,280 points of 1,310 and the odds value is 0.331.

### ROC and AUC of the predictive model

**Figure 4:**
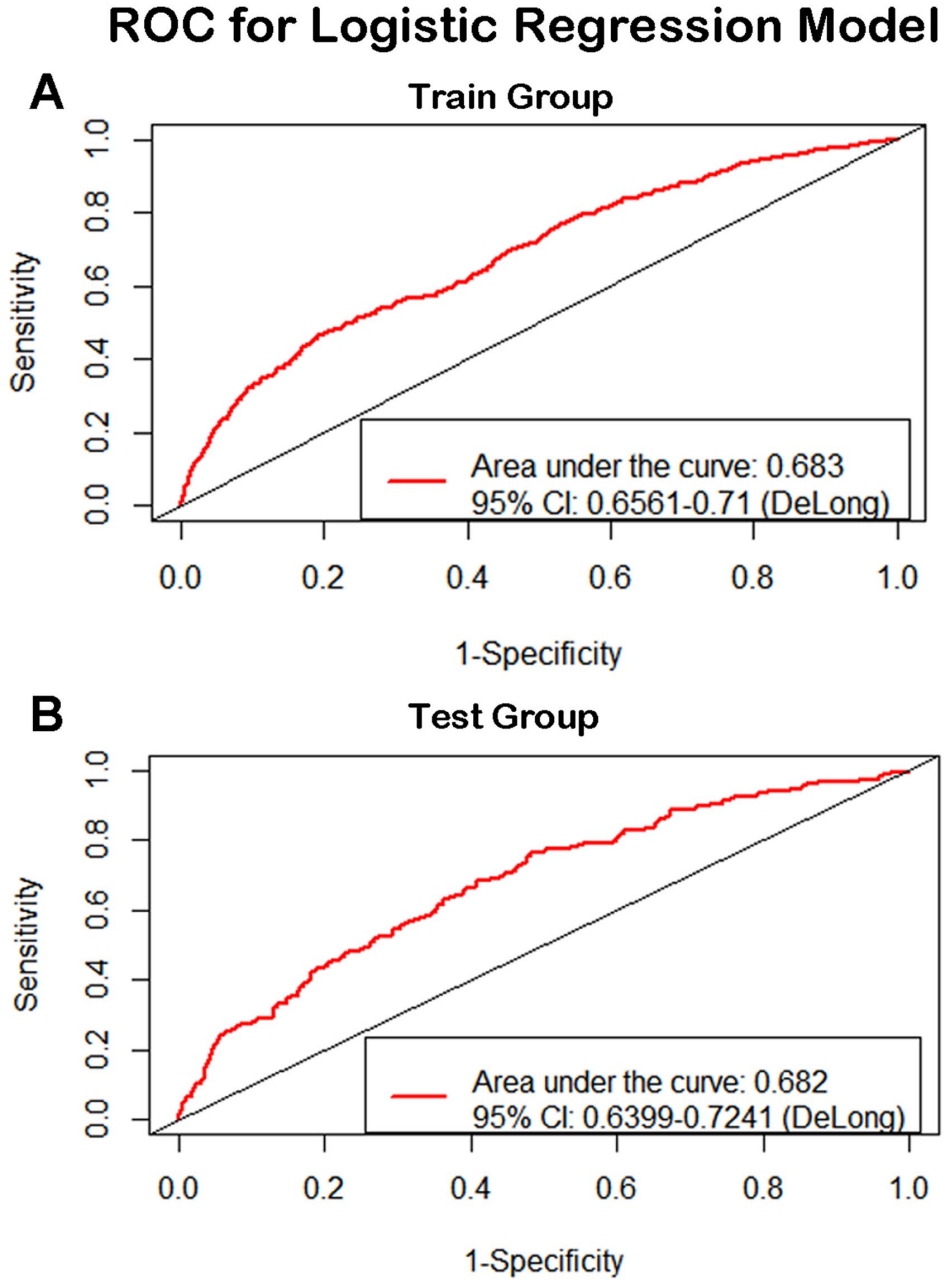
ROC for logistic regression model. The ROCs of the training and testing sets were plotted, with AUC values of 0.683 and 0.682 respectively. De Long tests showed that the 95%CI of the training set was 0.6561-0.71 and the testing set was 0.6399-0.7241.

**Figure 5:**
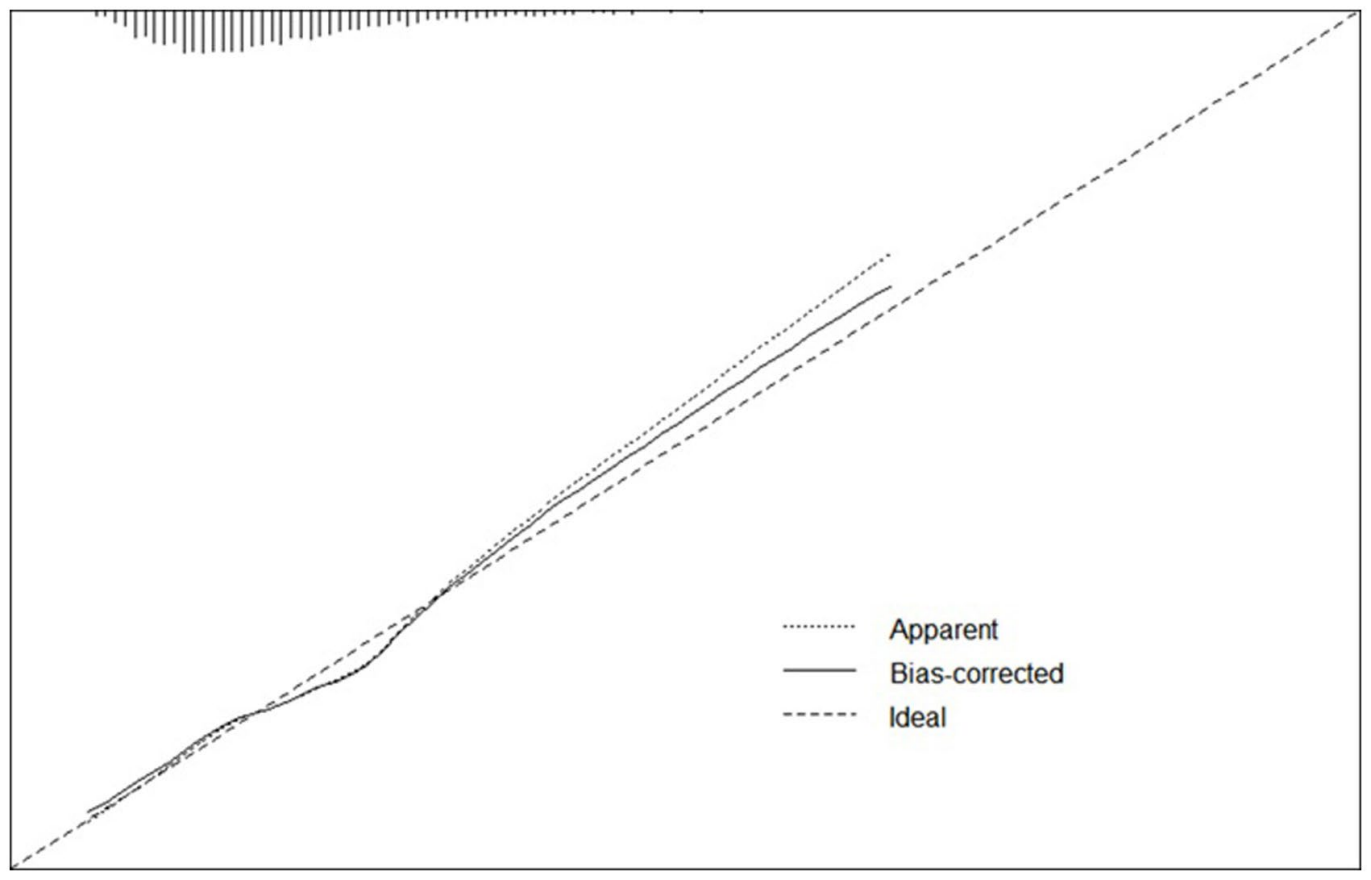
Calibration curve of logistic regression model. A calibration curve of the predictive model was plotted, with the mean absolute error of 0.012, mean squared error of 0.00018, and 0.9 quantile of absolute error of 0.024.

For continuous variables, age and height consistently demonstrated significant associations across single-factor comparisons, multiple linear regression, and logistic regression analyses. For categorical variables and subjective evaluations, such as gender, hypertension level, dietary habits, physical exercise duration, physical exercise history, and smoking history, significant associations were observed in both single-factor comparisons and logistic regression results. Although systolic pressure was found to have a significant relationship with cognitive function in all the analysis, the results were not consistent. We have performed additional analysis for explanation (Supplementary material 5) and elucidated the reasons in the discussion. In summary, gender, age, hypertension level, height, dietary habits, physical exercise duration, physical exercise history, and smoking history appear to be closely linked to cognitive decline in older individuals. These variables were used as risk and protective factors to develop a predictive model through logistic regression. The ROCs of the training and testing sets were plotted, with AUC values of 0.683 and 0.682, respectively (Figure 4). De Long tests showed that the 95%CI of the training set was 0.6561–0.71 and the testing set was 0.6399–0.7241 (Figure 4). A calibration curve of the predictive model was plotted (Figure 5), with the mean absolute error of 0.012, mean squared error of 0.00018, and 0.9 quantile of absolute error of 0.024.

The ROCs of the training and testing sets were plotted, with AUC values of 0.683 and 0.682, respectively. De Long tests showed that the 95%CI of the training set was 0.6561–0.71 and the testing set was 0.6399–0.7241.

A calibration curve of the predictive model was plotted, with the mean absolute error of 0.012, mean squared error of 0.00018, and 0.9 quantile of absolute error of 0.024.

### Comparison of the risk and protective factors between genders

Table 4 presented the gender differences in the risk and protective factors. The males were found to be older than females in this study (*p* < 0.05). The males also had greater height (*p* < 0.0001) and spent more time on physical exercise (*p* < 0.001). The number of physical-exercise years was greater in the males than females, although the difference was weakly significant (*p* < 0.1). The difference in smoking years between genders was highly significant (*p* < 0.0001). No significant differences were observed in hypertension level and dietary habit between genders.

**Table 4:** Gender differences in the risk and protective factors.

Characteristics	Male	Female	*p* value
	(*n* = 1,466)	(*n* = 2,242)	
Age (years)	72.39 (0.1507)	71.83 (0.1157)	0.0131^*^
Hypertension level^a^			
No hypertension	65 (4.43%)	114 (5.08%)	0.5932
Elevated	527 (35.95%)	839 (37.42%)	
High blood pressure stage 1	713 (48.64%)	1,064 (47.46%)	
High blood pressure stage 2	160 (10.91%)	222 (9.90%)	
High blood pressure crisis	1 (0.068%)	3 (0.13%)	
Height (cm)	168.7 (0.1700)	156.9 (0.1149)	<0.0001^****^
Dietary habit^a^			
Vegetable only	11 (0.75%)	31 (1.38%)	0.2666
More vegetables than meat	1 (0.068%)	5 (0.22%)	
Balanced diet	1,445 (98.57%)	2,190 (97.68%)	
More meat than vegetable	0 (0%)	1 (0.045%)	
Meat only	9 (0.61%)	15 (0.67%)	
Daily-physical-exercise duration (min)	36.56 (0.9251)	33.35 (0.8263)	0.0007^***^
Physical-exercise years	7.696 (0.1775)	7.194 (0.1358)	0.0578^#^
Smoking years	7.369 (0.4419)	0.03345 (0.02758)	<0.0001^****^

a Chi-square test is used for the comparison of the classification variables. ^#^*p* < 0.1, *p < 0.05, **p < 0.01, ***p < 0.001, ****p < 0.0001.

### Comparison of the risk and protective factors in the male and female participants

Table 5 presented the differences in risk and protective factors among the male participants. Out of 1,466 individuals, 1,285 displayed normal cognition, while 181 exhibited cognitive decline. Males with cognitive decline were significantly older (*p* < 0.0001) than those with normal cognition. Individuals with normal cognition were taller than those with cognitive decline (*p* < 0.01). The duration of physical exercise was longer in the normal group compared to the cognitive decline group (*p* < 0.05). Males with normal cognition had more years of physical exercise than those with cognitive decline (*p* < 0.01). Individuals with normal cognition had a longer history of smoking than those with cognitive decline (*p* < 0.01). No significant differences were found in hypertension levels and dietary habits between the groups.

**Table 5:** Comparison of the risk and protective factors in the male participants.

Characteristics	Total (*N* = 1,466)	Normal cognition (*n* = 1,285)	Cognitive decline (*n* = 181)	*p* value
Age (years)	72.39 (0.1507)	72.00 (0.1550)	75.11 (0.4839)	<0.0001^****^
Hypertension level^a^				
No hypertension	65 (4.433%)	54 (4.202%)	11 (6.077%)	0.5959
Elevated	527 (35.948%)	468 (36.42%)	59 (32.597%)	
High blood pressure stage 1	713 (48.636%)	617 (48.016%)	96 (53.039%)	
High blood pressure stage 2	160 (10.914%)	145 (11.284%)	15 (8.287%)	
High blood pressure crisis	1 (0.0682%)	1 (0.078%)	0 (0%)	
Height (cm)	168.7 (0.17)	168.9 (0.1805)	167.4 (0.4942)	0.0016^**^
Dietary habit^a^				
Vegetable only	11 (0.75%)	11 (0.0856%)	0 (0%)	0.8348
More vegetables than meat	1 (0.0682%)	1 (0.078%)	0 (0%)	
Balanced diet	1,445 (98.568%)	1,264 (98.366%)	181 (100%)	
More meat than vegetables	0 (0%)	0 (0%)	0 (0%)	
Meat only	9 (0.614%)	9 (0.7%)	0 (0%)	
Physical-exercise duration (min)	36.56 (0.9251)	37.05 (0.9861)	33.09 (2.663)	0.0402^*^
Physical-exercise years	7.696 (0.1775)	7.876 (0.19)	6.420 (0.487)	0.0028^**^
Smoking years	7.369 (0.4419)	7.846 (0.4836)	3.983 (0.9786)	0.0033^**^

a Chi-square test is used for the comparison of the classification variables. ^#^*p* < 0.1, *p < 0.05, **p < 0.01, ***p < 0.001, ****p < 0.0001.

Table 6 illustrated the differences in risk and protective factors among the female participants. Of the 2,242 individuals, 1,763 displayed normal cognition, while 479 showed cognitive decline. Females with cognitive decline were significantly older (*p* < 0.0001) than those with normal cognition. Females with cognitive decline had a higher proportion of high blood pressure (*p* < 0.0001). Individuals with normal cognition were taller than those with cognitive decline (*p* < 0.0001). Significant differences were observed in dietary habits among female participants (*p* < 0.05). The proportion of females with cognitive decline following a ‘balanced diet’ was lower, and they consumed more vegetables in their diet. No significant differences were observed in ‘physical-exercise duration,’ ‘physical-exercise years,’ and ‘smoking years.’

**Table 6:** Comparison of the risk and protective factors in the female participants.

Characteristics	Total (*N* = 2,242)	Normal cognition (*n* = 1,763)	Cognitive decline (*n* = 479)	*p* value
Age (years)	71.83 (0.1157)	71.17 (0.1194)	74.28 (0.2908)	<0.0001^****^
Hypertension level^a^				
No hypertension	114 (5.085%)	94 (5.332%)	20 (4.175%)	<0.0001^****^
Elevated	839 (37.422%)	699 (39.648%)	140 (29.228%)	
High blood pressure stage 1	1,064 (47.458%)	791 (44.867%)	273 (56.994%)	
High blood pressure stage 2	222 (9.902%)	178 (10.096%)	44 (9.186%)	
High blood pressure crisis	3 (0.134%)	1 (0.057%)	2 (0.418%)	
Height (cm)	156.9 (0.1149)	157.2 (0.1284)	155.8 (0.2514)	<0.0001^****^
Dietary habit^a^				
Vegetable only	31 (1.383%)	18 (1.021%)	13 (2.714%)	0.0254^*^
More vegetables than meat	5 (0.223%)	3 (0.17%)	2 (0.418%)	
Balanced diet	2,190 (97.681%)	1727 (97.958%)	463 (96.66%)	
More meat than vegetables	1 (0.0446%)	1 (0.057%)	0 (0%)	
Meat only	15 (0.669%)	14 (0.794%)	1 (0.209%)	
Physical-exercise duration (min)	33.35 (0.8263)	31.54 (0.8116)	35.33 (2.536)	0.4382
Physical-exercise years	7.194 (0.1358)	7.199 (0.15)	7.175 (0.3156)	0.4302
Smoking years	0.0335 (0.0276)	0.0425 (0.0351)	0 (0)	0.4614

a Chi-square test is used for the comparison of the classification variables. ^#^*p* < 0.1, *p < 0.05, **p < 0.01, ***p < 0.001, ****p < 0.0001.

## Discussion

Numerous factors in daily life are closely linked to the cognitive health of older adults. Our research found that individuals with normal cognition and those with cognitive decline were distributed differently on the scale by PCA. This suggests that cognitive decline is not a random occurrence, but rather the result of complex factors in daily life. Upon further analysis, we identified several potential risk and protective factors associated with cognitive decline in older adults. These include age, dietary habits, hypertension, height, daily physical exercise duration, years of physical exercise, and years of smoking. Most of these factors, which encompass physical condition, disease, and lifestyle, are modifiable or preventable. Therefore, appropriate interventions could be performed to potentially improve and maintain brain function in older adults, thereby reducing the risk of cognitive decline (Kivipelto et al., 2018).

Age, the only unmodifiable factor among the results mentioned, is positively associated with cognitive decline. The aging process in the brain differs from that in other organs of the human body, as neurons are highly differentiated and incapable of regeneration during the lifespan (Isaev et al., 2019; Isaev et al., 2013). Aging can significantly impair neurogenesis, further damaging the cognitive function of older adults (Isaev et al., 2019). While age cannot be changed, recent findings on Neural Stem Cells (NSCs) suggest that brain aging could be intervened. NSC treatment could maintain neurogenesis at a youthful state during aging, offering a promising approach to preventing age-related cognitive decline (Isaev et al., 2019).

Dietary and nutritional elements are factors which can be adjusted to delay or prevent cognitive decline. It is widely recognized that an unhealthy diet is a major risk factor for cognitive decline (Norton et al., 2014). For instance, after Japan transitioned from a traditional dietary pattern to a Western one, the Alzheimer’s Disease (AD) rate increased from 1% in 1985 to 7% in 2008 (Grant, 2016). So, what constitutes a diet which is beneficial for cognitive function? People are often advised to consume more vegetables and fruits because these plant foods are low in calories and high in fiber, which is beneficial to the body and could reduce the chances of moderate or severe cognitive decline (Yuan et al., 2019). Therefore, it seems reasonable to suggest that a vegan diet could prevent cognitive decline by potentially exerting neuroprotective effects. However, there is no solid evidence to confirm that a vegetarian diet can lower the risk of cognitive decline more than a diet that includes meat. A study conducted by Giem et al. (1993), which included 2,984 participants, investigated the relationship between dietary patterns (vegetarian and meat-eating) and the incidence of dementia, reporting no significant differences between the two groups. From our perspective, a vegetarian diet does not decrease the risk of cognitive decline in older individuals. According to our findings, a diet with a very high proportion of vegetables can actually increase the risk of cognitive decline. Therefore, we suggest increasing the proportion of meat in the diet to ensure a diverse intake of nutrients from food. The combination of nutrients can have synergistic and/or antagonistic effects beyond single components, which may have antioxidant and anti-inflammatory effects on the brain and improve cognitive function (Dominguez et al., 2021; Jacobs and Orlich, 2014).

Physical activity is favored among older adults, as many believe that it can slow down body aging. Physical activity is also thought as an effective way to enhance cognitive function. Studies suggest that increasing physical activity can lower dementia risk by 3% (Livingston et al., 2017; Liang et al., 2020). Evidence shows that the progression of cognitive decline can be hindered or slowed by physical activity (Vancampfort et al., 2020). Older individuals who engage in more physical exercise are more likely to maintain cognitive function and reduce dementia risk compared to those who are sedentary (Livingston et al., 2017; Sofi et al., 2011). Furthermore, the progression from mild cognitive impairment (MCI) to dementia can be delayed by physical activity (Nuzum et al., 2020). This study showed a close relationship between physical activity/exercise and cognitive function. On one hand, individuals with a longer history of exercise are less likely to experience cognitive decline. On the other hand, prolonged exercise duration may increase the risk of cognitive decline. The findings suggest that maintaining physical activity/exercise can help preserve the cognitive function of older people. However, the duration of exercise should be regulated to prevent overloading the body. It is well-known that heart function diminishes with aging. Therefore, the heart function of the older cannot sustain extended periods of exercise, which could lead to brain hypoxia. Based on our findings, although the mean values of exercise duration between the two groups were close to each other, the samples with cognitive decline still demonstrated a longer exercise session. Thus, the older individual should pay attention to their body condition in a physical exercise, and we recommend that leisure sports such as walking and jogging are good choices for the older rather than the vigorous exercises. We also propose that each exercise session should not exceed 30 min according to the results from the present study.

Vascular conditions, particularly hypertension, play a significant role in the progression of cognitive decline and dementia (Santisteban et al., 2023). Postmortem examinations have revealed neurovascular pathology in over 50% of Alzheimer’s disease (AD) patients (Azarpazhooh et al., 2018; Santisteban and Iadecola, 2018). If the genetic predisposition for dementia is not taken into account, hypertension during midlife can increase the likelihood of dementia in later life (McGrath et al., 2017; Littlejohns et al., 2023). It is well understood that chronic hypertension can lead to neurovascular remodeling, reducing cerebral blood flow and ultimately contributing to tissue damage and cognitive decline. This study further substantiates that hypertension is a contributing factor to cognitive decline. Moreover, the risk of cognitive decline is directly proportional to the level of hypertension. It is crucial for older adults to monitor their blood pressure regularly, and lifelong treatment is necessary if hypertension is diagnosed. The study revealed an intriguing finding that systolic pressure was positively correlated with the cognitive function based on the results from multiple linear regression and logistic regression, while the group with cognitive decline demonstrated a higher systolic pressure than the group with normal cognition. These results were not contradictory. It is important to note that hypertension is a pathological condition and diagnosed not only by systolic pressure but also by considering diastolic pressure. A higher blood pressure within the physiological range does not exert significant impact on the body. Thus, hypertension level is more effective in assessing the cardiovascular condition of a patient. It is worth noting that blood pressure may decrease or even approach normal levels before the onset of dementia and reaches its lowest levels in advanced stages of the dementia (Kennelly et al., 2009; Stewart et al., 2009). We performed simple linear regressions between MMSE and systolic pressure to further validate previous findings. Interestingly, we observed a significant positive correlation between MMSE scores and systolic pressure in the older adults with cognitive decline, while non-significant negative correlation was noted in the normal group and the overall sample (Supplementary material 5). We assume that regulation of blood pressure could be impaired during the biological progression of dementia, leading to the reduced capacity to maintain the blood flow. This explains why the systolic pressure decreases parallel with MMSE scores after the onset of cognitive decline. Collectively, systolic pressure could be used to assess the degree of dementia rather than as a predictor. People should pay great attention to their hypertension level before the onset of cognitive impairment.

It is widely recognized that smoking is a detrimental habit, linked to a multitude of diseases such as lung cancer, hypertension, hyperlipidemia, pneumonia, and chronic obstructive pulmonary disease. It seems reasonable to assume that smoking could lead to cognitive decline due to neurovascular lesions. However, this study suggests a contrasting narrative: older adults with a longer history of smoking appear to have a lower risk of cognitive decline. This unexpected finding could be attributed to the neurological effects of nicotine, which has been shown to enhance cognitive function at typical smoking levels (Waisman Campos et al., 2016). Nicotine exerts its influence by interacting with nicotinic acetylcholine receptors (nAChRs), ligand-gated ion channels, which are formed by various pentameric combinations (Clader and Wang, 2005). These combinations arrange a central pore that allows the passage of sodium, potassium, and calcium ions (Dani and Bertrand, 2007). The majority of neuronal nAChRs in the brain are excitatory and fast-acting, modulating the release of other neurotransmitters including acetylcholine (ACh), dopamine (DA), serotonin, glutamate, GABA, and norepinephrine (Clader and Wang, 2005; Dani and Bertrand, 2007). The prefrontal cortex and hippocampus, regions with a high concentration of nAChRs, are associated with the cognitive effects of nicotine (Wallace and Bertrand, 2013; Kutlu and Gould, 2015). Cognitive function is enhanced due to improvements in signal-to-noise ratios or the facilitation of synaptic plasticity in specific neural circuits within these two regions (Wallace and Bertrand, 2013; Couey et al., 2007). The beneficial effect from nicotine is even supported by molecular evidence. In healthy individuals of sleep deprivation, nicotine could increase the phosphorylation of calmodulin-dependent protein kinase II to help cell proliferation and synaptic plasticity, leading to the improvement of memory impairment (Alhowail, 2021). Exposure to nicotine could also enhance the hippocampus-dependent learning and memory (Kutlu and Gould, 2016). Nicotine could attenuate cognitive impairment of AD patients by increasing Akt activity and stimulating Pi3k/Akt signaling pathway (Alhowail, 2021). It is important to note that we do not advocate for older adults to take up smoking as a means to improve cognitive function. This is due to the other toxic effects of cigarettes, which can ultimately lead to diseases in the vascular and respiratory systems.

This study revealed an interesting result: height is positively associated with cognition in older adults, which aligns with findings from previous studies (Amin and Fletcher, 2022; Case and Paxson, 2008). Additionally, height is considered a protective factor against age-related cognitive impairment (Guven and Lee, 2015). Surprisingly, genetic factors do not account for the relationship between height and cognition, as demonstrated by twin studies (Amin and Fletcher, 2022). The association between height and cognition may be influenced by social and environmental factors. Height is often biasedly linked to positive attributes such as intelligence, competence, attractiveness, and social status (Amin and Fletcher, 2022). Consequently, taller individuals tend to receive more favorable treatment from a young age. As a result, taller people are likely to have better education, higher income, and greater access to medical resources, all of which positively impact cognition. These, altogether, exert positive influence on cognition. Diet during early childhood could play a crucial role in determining height. Nutritional adequacy during youth may have lasting effects on cognitive abilities throughout the lifespan. Notably, taller children often demonstrate enhanced cognitive abilities, which could be attributed to sufficient nutrient intake (Guo and Song, 2023). Collectively, the associations between height and cognition are extensive, resulting from a combination of social and environmental factors.

The differences between males and females span various aspects, including behavior, thought patterns, emotions, and cognition. Our study revealed a gender bias in cognitive decline, with females more likely to exhibit cognitive decline in their later years. This could be attributed to the differing brain structures between the two genders (Li and Singh, 2014). Studies based on Magnetic Resonance Imaging (MRI) have shown that the volumes of the amygdala and thalamus in males are larger than those in females (Bramen et al., 2011; Koolschijn and Crone, 2013; Neufang et al., 2009), while females have a larger hippocampus (Neufang et al., 2009; Giedd et al., 1996). It is worth noting that amygdala contains high concentrations of androgen receptors (Clark et al., 1988), while hippocampus have high number of estrogen receptors (Morse et al., 1986). As we know, the estrogen level in females drastically decreases with aging, impacting hippocampal function and contributing to cognitive decline. In contrast, the androgen level in males decreases gradually with aging, potentially helping to maintain cognitive function in later years. We compared the risk and protective factors among participants of both genders, respectively. Our findings revealed that men with normal cognition tend to exercise for longer durations, a result that contradicts the findings from the entire participant group. This discrepancy could be attributed to the physiological fact that males typically have stronger cardiorespiratory functions than females. Furthermore, the cognition of older females is more likely to be influenced by hypertension, which could be associated with the rapid decrease in estrogen levels. Studies have shown that the absence of ovarian-produced E2 and P4 can accelerate the development of cardiovascular diseases (Gersh et al., 2024). The resulting decline in vascular function could lead to brain damage and subsequent cognitive decline. Beyond genetic differences, our results indicated that men have a longer history of exercise than women, and most women do not smoke. We also discovered a significant difference in the dietary habits of older females between the two groups, but not in males. These lifestyle differences could also contribute to the observed cognitive differences between the genders.

We developed a predictive model using logistic regression, based on the most significant protective and risk factors. The AUC values for the training and testing sets were 0.683 and 0.682, respectively. Although the AUC value of an effective predictive model is thought to be over 0.7, we still believe that the present model is acceptable because the value approaches the threshold. This further validates the protective and risk factors identified in our study.

We must acknowledge some limitations of the present study. Although the samples were not selected with bias, the older individuals were recruited from a small community in Shanghai, which may not accurately represent the older adult worldwide. Additionally, this research only analyzed factors related to cognitive decline using data from basic clinical measurements and daily behaviors. No genetic factors or serum biomarkers were included in building the model or to further elucidate the mechanisms of age-related cognitive impairment. Thus, the AUC value of the predictive model was not high enough to meet the standard as an effective diagnostic tool. We believe that a more robust predictive model could be developed if genetic factors and serum biomarkers are taken into consideration.

## Conclusion

**Figure 6:**
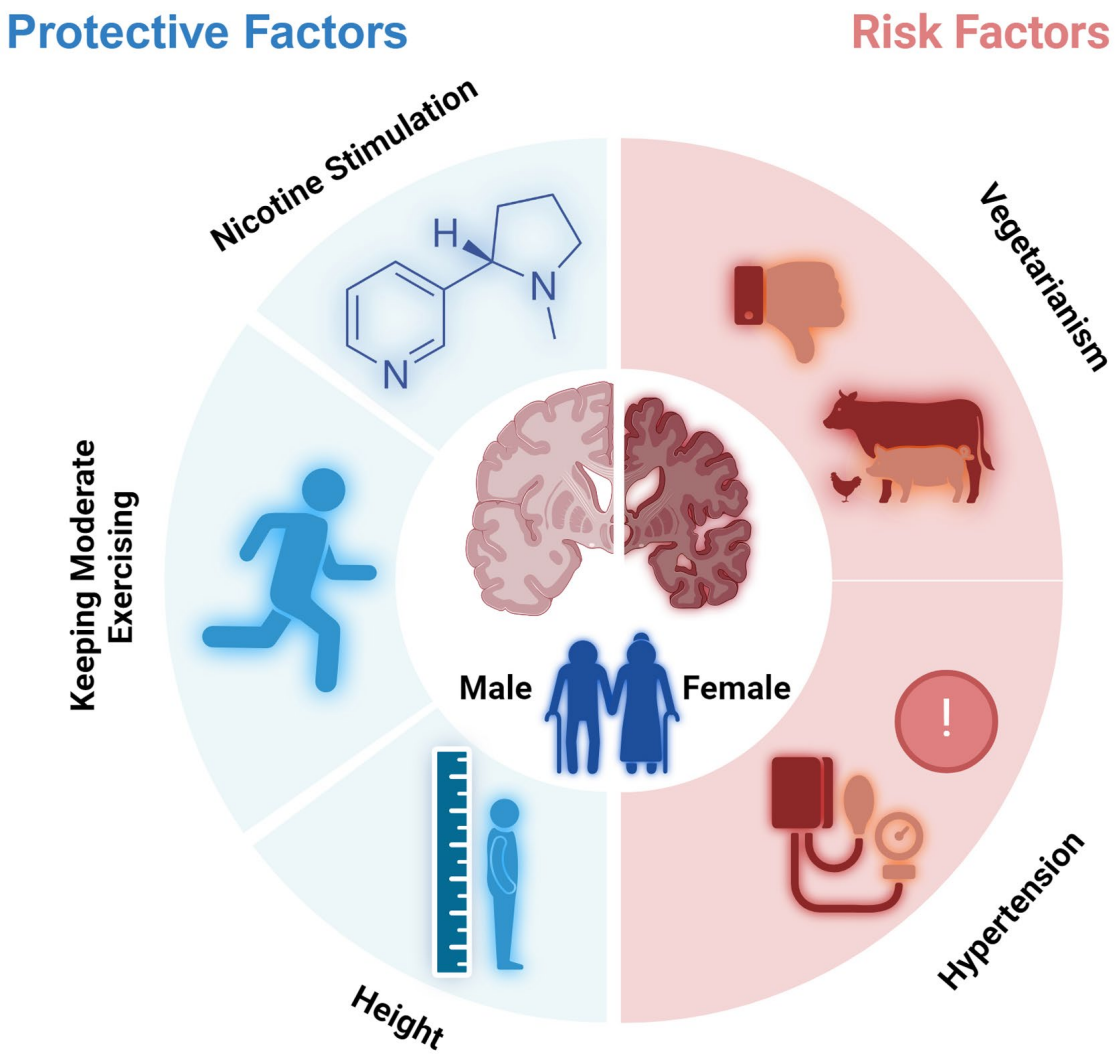
Protective and risk factors for cognitive decline of older adults (Created with BioRender.com).

In conclusion, the protective factors of cognitive decline for older people were male gender, height, keeping moderate exercising, and nicotine stimulation, and the risk factors included age, female gender, vegetarianism, hypertension, and over-exercise (Figure 6). Except for the genetic factor, differences in lifestyle, such as smoking and exercise habits, may contribute to the observed differences in cognitive function between genders.

## Data Availability

The raw data supporting the conclusions of this article will be made available by the authors, without undue reservation.

## References

[ref1] Alhowail A. (2021). Molecular insights into the benefits of nicotine on memory and cognition (review). Mol. Med. Rep. 23:398. doi: 10.3892/mmr.2021.12037, PMID: 33786606 PMC8025477

[ref2] Amin V. Fletcher J. M. (2022). What is driving the relationship between height and cognition? Evidence from the twins early development study. Econ. Hum. Biol. 47:101174. doi: 10.1016/j.ehb.2022.101174, PMID: 36027762 PMC9872705

[ref3] Arevalo-Rodriguez I. Smailagic N. Roqué-Figuls M. Ciapponi A. Sanchez-Perez E. Giannakou A.. (2021). Mini-mental state examination (MMSE) for the early detection of dementia in people with mild cognitive impairment (MCI). Cochrane Database Syst. Rev. 2021:CD010783. doi: 10.1002/14651858.CD010783.pub3, PMID: 34313331 PMC8406467

[ref4] Azarpazhooh M. R. Avan A. Cipriano L. E. Munoz D. G. Sposato L. A. Hachinski V. (2018). Concomitant vascular and neurodegenerative pathologies double the risk of dementia. Alzheimers Dement. 14, 148–156. doi: 10.1016/j.jalz.2017.07.755, PMID: 28974416

[ref5] Beard J. R. Officer A. de Carvalho I. A. Sadana R. Pot A. M. Michel J. P.. (2016). The world report on ageing and health: a policy framework for healthy ageing. Lancet 387, 2145–2154. doi: 10.1016/S0140-6736(15)00516-4, PMID: 26520231 PMC4848186

[ref6] Bramen J. E. Hranilovich J. A. Dahl R. E. Forbes E. E. Chen J. Toga A. W.. (2011). Puberty influences medial temporal lobe and cortical gray matter maturation differently in boys than girls matched for sexual maturity. Cereb. Cortex 21, 636–646. doi: 10.1093/cercor/bhq137, PMID: 20713504 PMC3041011

[ref7] Case A. Paxson C. (2008). Stature and status: height, ability, and labor market outcomes. J. Polit. Econ. 116, 499–532. doi: 10.1086/589524, PMID: 19603086 PMC2709415

[ref8] Clader J. W. Wang Y. (2005). Muscarinic receptor agonists and antagonists in the treatment of Alzheimer's disease. Curr. Pharm. Des. 11, 3353–3361. doi: 10.2174/138161205774370762, PMID: 16250841

[ref9] Clark A. S. Maclusky N. J. Goldman-Rakic P. S. (1988). Androgen binding and metabolism in the cerebral cortex of the developing rhesus monkey. Endocrinology 123, 932–940. doi: 10.1210/endo-123-2-932, PMID: 3260856

[ref10] Couey J. J. Meredith R. M. Spijker S. Poorthuis R. B. Smit A. B. Brussaard A. B.. (2007). Distributed network actions by nicotine increase the threshold for spike-timing-dependent plasticity in prefrontal cortex. Neuron 54, 73–87. doi: 10.1016/j.neuron.2007.03.006, PMID: 17408579

[ref11] Crum R. M. Anthony J. C. Bassett S. S. Folstein M. F. (1993). Population-based norms for the Mini-mental state examination by age and educational level. JAMA 269, 2386–2391. doi: 10.1001/jama.1993.03500180078038 8479064

[ref12] Dani J. A. Bertrand D. (2007). Nicotinic acetylcholine receptors and nicotinic cholinergic mechanisms of the central nervous system. Annu. Rev. Pharmacol. Toxicol. 47, 699–729. doi: 10.1146/annurev.pharmtox.47.120505.105214 17009926

[ref13] Dominguez L. J. Veronese N. Vernuccio L. Catanese G. Inzerillo F. Salemi G.. (2021). Nutrition, physical activity, and other lifestyle factors in the prevention of cognitive decline and dementia. Nutrients 13:4080. doi: 10.3390/nu13114080, PMID: 34836334 PMC8624903

[ref14] Gersh F. O'Keefe J. H. Elagizi A. Lavie C. J. Laukkanen J. A. (2024). Estrogen and cardiovascular disease. Prog. Cardiovasc. Dis. 84, 60–67. doi: 10.1016/j.pcad.2024.01.015, PMID: 38272338

[ref15] Giedd J. N. Snell J. W. Lange N. Rajapakse J. C. Casey B. Kozuch P. L.. (1996). Quantitative magnetic resonance imaging of human brain development: ages 4–18. Cereb. Cortex 6, 551–559. doi: 10.1093/cercor/6.4.551, PMID: 8670681

[ref16] Giem P. Beeson W. L. Fraser G. E. (1993). The incidence of dementia and intake of animal products: preliminary findings from the Adventist health study. Neuroepidemiology 12, 28–36. doi: 10.1159/000110296, PMID: 8327020

[ref17] Grant W. B. (2016). Using multicountry ecological and observational studies to determine dietary risk factors for Alzheimer's disease. J. Am. Coll. Nutr. 35, 476–489. doi: 10.1080/07315724.2016.1161566 27454859

[ref18] Guo J. Song S. (2023). Associations of height loss with cognitive decline and incident dementia in adults aged 50 years and older. J. Gerontol. A Biol. Sci. Med. Sci. 78, 1445–1452. doi: 10.1093/gerona/glad054, PMID: 36754370

[ref19] Guven C. Lee W. S. (2015). Height, aging and cognitive abilities across Europe. Econ. Hum. Biol. 16, 16–29. doi: 10.1016/j.ehb.2013.12.005, PMID: 24485906

[ref20] Isaev N. K. Stelmashook E. V. Genrikhs E. E. (2019). Neurogenesis and brain aging. Rev. Neurosci. 30, 573–580. doi: 10.1515/revneuro-2018-0084 30763272

[ref21] Isaev N. Stelmashook E. Stelmashook N. Sharonova I. Skrebitsky V. (2013). Brain aging and mitochondria-targeted plastoquinone antioxidants of SkQ-type. Biochem. Mosc. 78, 295–300. doi: 10.1134/S0006297913030127, PMID: 23586724

[ref22] Iwashyna T. J. Ely E. W. Smith D. M. Langa K. M. (2010). Long-term cognitive impairment and functional disability among survivors of severe sepsis. JAMA 304, 1787–1794. doi: 10.1001/jama.2010.1553, PMID: 20978258 PMC3345288

[ref23] Jacobs D. R. Jr. Orlich M. J. (2014). Diet pattern and longevity: do simple rules suffice? A commentary. Am. J. Clin. Nutr. 100, 313S–319S. doi: 10.3945/ajcn.113.071340, PMID: 24871470 PMC4144105

[ref24] Kennelly S. P. Lawlor B. A. Kenny R. A. (2009). Blood pressure and dementia - a comprehensive review. Ther. Adv. Neurol. Disord. 2, 241–260. doi: 10.1177/1756285609103483, PMID: 21179532 PMC3002634

[ref25] Kivipelto M. Mangialasche F. Ngandu T. (2018). Lifestyle interventions to prevent cognitive impairment, dementia and Alzheimer disease. Nat. Rev. Neurol. 14, 653–666. doi: 10.1038/s41582-018-0070-3, PMID: 30291317

[ref26] Koolschijn P. C. M. Crone E. A. (2013). Sex differences and structural brain maturation from childhood to early adulthood. Dev. Cogn. Neurosci. 5, 106–118. doi: 10.1016/j.dcn.2013.02.003, PMID: 23500670 PMC6987760

[ref27] Kutlu M. G. Gould T. J. (2015). Nicotinic receptors, memory, and hippocampus. Curr. Top. Behav. Neurosci. 23, 137–163. doi: 10.1007/978-3-319-13665-3_6, PMID: 25655890

[ref28] Kutlu M. G. Gould T. J. (2016). Effects of drugs of abuse on hippocampal plasticity and hippocampus-dependent learning and memory: contributions to development and maintenance of addiction. Learn. Mem. 23, 515–533. doi: 10.1101/lm.042192.116, PMID: 27634143 PMC5026208

[ref29] Li R. Singh M. (2014). Sex differences in cognitive impairment and Alzheimer’s disease. Front. Neuroendocrinol. 35, 385–403. doi: 10.1016/j.yfrne.2014.01.002, PMID: 24434111 PMC4087048

[ref30] Liang J.-h. Lu L. Li J.-y. Qu X.-y. Li J. Qian S.. (2020). Contributions of modifiable risk factors to dementia incidence: a Bayesian network analysis. J. Am. Med. Dir. Assoc. 21, 1592–1599.e13. e13. doi: 10.1016/j.jamda.2020.04.006, PMID: 32563753

[ref31] Littlejohns T. J. Collister J. A. Liu X. Clifton L. Tapela N. M. Hunter D. J. (2023). Hypertension, a dementia polygenic risk score, APOE genotype, and incident dementia. Alzheimers Dement. 19, 467–476. doi: 10.1002/alz.12680, PMID: 35439339

[ref32] Livingston G. Sommerlad A. Orgeta V. Costafreda S. G. Huntley J. Ames D.. (2017). Dementia prevention, intervention, and care. Lancet 390, 2673–2734. doi: 10.1016/S0140-6736(17)31363-6, PMID: 28735855

[ref33] McGrath E. R. Beiser A. S. DeCarli C. Plourde K. L. Vasan R. S. Greenberg S. M.. (2017). Blood pressure from mid-to late life and risk of incident dementia. Neurology 89, 2447–2454. doi: 10.1212/WNL.0000000000004741, PMID: 29117954 PMC5729797

[ref34] McKhann G. M. Knopman D. S. Chertkow H. Hyman B. T. Jack C. R. Jr. Kawas C. H.. (2011). The diagnosis of dementia due to Alzheimer's disease: recommendations from the National Institute on Aging-Alzheimer's Association workgroups on diagnostic guidelines for Alzheimer's disease. Alzheimers Dement. 7, 263–269. doi: 10.1016/j.jalz.2011.03.005, PMID: 21514250 PMC3312024

[ref35] Morse J. K. Scheff S. W. DeKosky S. T. (1986). Gonadal steroids influence axon sprouting in the hippocampal dentate gyrus: a sexually dimorphic response. Exp. Neurol. 94, 649–658. doi: 10.1016/0014-4886(86)90244-X, PMID: 3780911

[ref36] Murman D. L. (2015). The impact of age on cognition. Semin. Hear. 36, 111–121. doi: 10.1055/s-0035-1555115, PMID: 27516712 PMC4906299

[ref37] Neufang S. Specht K. Hausmann M. Gunturkun O. Herpertz-Dahlmann B. Fink G. R.. (2009). Sex differences and the impact of steroid hormones on the developing human brain. Cereb. Cortex 19, 464–473. doi: 10.1093/cercor/bhn100, PMID: 18550597

[ref38] Norton S. Matthews F. E. Barnes D. E. Yaffe K. Brayne C. (2014). Potential for primary prevention of Alzheimer's disease: an analysis of population-based data. Lancet Neurol. 13, 788–794. doi: 10.1016/S1474-4422(14)70136-X, PMID: 25030513

[ref39] Nuzum H. Stickel A. Corona M. Zeller M. Melrose R. J. Wilkins S. S. (2020). Potential benefits of physical activity in MCI and dementia. Behav. Neurol. 2020, 1–10. doi: 10.1155/2020/7807856, PMID: 32104516 PMC7037481

[ref40] Prince M. Ali G. C. Guerchet M. Prina A. M. Albanese E. Wu Y. T. (2016). Recent global trends in the prevalence and incidence of dementia, and survival with dementia. Alzheimers Res. Ther. 8:23. doi: 10.1186/s13195-016-0188-8, PMID: 27473681 PMC4967299

[ref41] Prince M. J. Wu F. Guo Y. Gutierrez Robledo L. M. O'Donnell M. Sullivan R.. (2015). The burden of disease in older people and implications for health policy and practice. Lancet 385, 549–562. doi: 10.1016/S0140-6736(14)61347-7, PMID: 25468153

[ref42] Santisteban M. M. Iadecola C. (2018). Hypertension, dietary salt and cognitive impairment. J. Cereb. Blood Flow Metab. 38, 2112–2128. doi: 10.1177/0271678X18803374, PMID: 30295560 PMC6282225

[ref43] Santisteban M. M. Iadecola C. Carnevale D. (2023). Hypertension, neurovascular dysfunction, and cognitive impairment. Hypertension 80, 22–34. doi: 10.1161/HYPERTENSIONAHA.122.18085, PMID: 36129176 PMC9742151

[ref44] Scheltens P. de B. Kivipelto M. Holstege H. Chételat G. Teunissen C. E.. (2021). Alzheimer's disease. Lancet 397, 1577–1590. doi: 10.1016/S0140-6736(20)32205-4, PMID: 33667416 PMC8354300

[ref45] Shim Y. S. Yang D. W. Kim H.-J. Park Y. H. Kim S. (2017). Characteristic differences in the mini-mental state examination used in Asian countries. BMC Neurol. 17:141. doi: 10.1186/s12883-017-0925-z, PMID: 28732484 PMC5521149

[ref46] Sofi F. Valecchi D. Bacci D. Abbate R. Gensini G. F. Casini A.. (2011). Physical activity and risk of cognitive decline: a meta-analysis of prospective studies. J. Intern. Med. 269, 107–117. doi: 10.1111/j.1365-2796.2010.02281.x, PMID: 20831630

[ref47] Stewart R. Xue Q. L. Masaki K. Petrovitch H. Ross G. W. White L. R.. (2009). Change in blood pressure and incident dementia: a 32-year prospective study. Hypertension 54, 233–240. doi: 10.1161/HYPERTENSIONAHA.109.128744, PMID: 19564551 PMC3136040

[ref48] Vancampfort D. Solmi M. Firth J. Vandenbulcke M. Stubbs B. (2020). The impact of pharmacologic and nonpharmacologic interventions to improve physical health outcomes in people with dementia: a meta-review of meta-analyses of randomized controlled trials. J. Am. Med. Dir. Assoc. 21, 1410–1414.e2. e2. doi: 10.1016/j.jamda.2020.01.010, PMID: 32085951

[ref49] Vaupel J. W. (2010). Biodemography of human ageing. Nature 464, 536–542. doi: 10.1038/nature08984, PMID: 20336136 PMC4010874

[ref50] Waisman Campos M. Serebrisky D. Mauricio Castaldelli-Maia J. (2016). Smoking and cognition. Curr. Drug Abuse Rev. 9, 76–79. doi: 10.2174/1874473709666160803101633 27492358

[ref51] Wallace T. Bertrand D. (2013). Importance of the nicotinic acetylcholine receptor system in the prefrontal cortex. Biochem. Pharmacol. 85, 1713–1720. doi: 10.1016/j.bcp.2013.04.001, PMID: 23628449

[ref52] Xu J. Gou S. Huang X. Zhang J. Zhou X. Gong X.. (2023). Uncovering the impact of Aggrephagy in the development of Alzheimer's disease: insights into diagnostic and therapeutic approaches from machine learning analysis. Curr. Alzheimer Res. 20, 618–635. doi: 10.2174/0115672050280894231214063023, PMID: 38141185

[ref53] Yaffe K. Laffan A. M. Harrison S. L. Redline S. Spira A. P. Ensrud K. E.. (2011). Sleep-disordered breathing, hypoxia, and risk of mild cognitive impairment and dementia in older women. JAMA 306, 613–619. doi: 10.1001/jama.2011.1115, PMID: 21828324 PMC3600944

[ref54] Yuan C. Fondell E. Bhushan A. Ascherio A. Okereke O. I. Grodstein F.. (2019). Long-term intake of vegetables and fruits and subjective cognitive function in US men. Neurology 92, e63–e75. doi: 10.1212/WNL.0000000000006684, PMID: 30464030 PMC6336164

