# Supplementary material for: Protective and risk factors in daily life associated with cognitive decline of older adults
Source: Front Aging Neurosci. 2025 Feb 26;17:1496677. doi: 10.3389/fnagi.2025.1496677 (PMC11897038; doi:10.3389/fnagi.2025.1496677)
Supplement: Supplementary file 1 [file Table_1.DOCX]

| **gender** |  |
| --- | --- |
| male | □ |
| female | □ |
| **age** |  |
| **blood pressure** |  |
| **self-assessment of health** |  |
| dissatisfactory | □ |
| not quite satisfactory | □ |
| basically satisfactory | □ |
| satisfactory | □ |
| **dietary habit** |  |
| only eat vegetable dishes for meal | □ |
| more vegetable dishes than meat for a meal | □ |
| balanced diet | □ |
| more meat dishes than vegetable for a meal | □ |
| only eat meat dishes for meal | □ |
| **physical-exercise** |  |
| never do exercise | □ |
| do exercise occasionally | □ |
| more than once a week | □ |
| every day | □ |
| **smoking** |  |
| never smoke | □ |
| used to smoke, not smoke now | □ |
| smoke now, but not every day | □ |
| smoke everyday | □ |
| **drinking** |  |
| never drink | □ |
| drink occasionally | □ |
| always drink but not every day | □ |
| drink everyday | □ |

Questionnaire in the survey
