# Supplementary material for: Protective and risk factors in daily life associated with cognitive decline of older adults
Source: Front Aging Neurosci. 2025 Feb 26;17:1496677. doi: 10.3389/fnagi.2025.1496677 (PMC11897038; doi:10.3389/fnagi.2025.1496677)
Supplement: Supplementary file 2 [file Table_2.DOCX]

Information on the classification and assignment of variables

| **Characteristics** | **Value assignment** |
| --- | --- |
|  |  |
| gender |  |
| male | 1 |
| female | 0 |
| hypertension level^a^ |  |
| no hypertension | 0 |
| elevated | 1 |
| high blood pressure stage 1 | 2 |
| high blood pressure stage 2 | 3 |
| high blood pressure crisis | 4 |
| self-assessment of health |  |
| dissatisfactory | 0 |
| not quite satisfactory | 1 |
| basically satisfactory | 2 |
| satisfactory | 3 |
| dietary habit |  |
| vegetable only | 0 |
| more vegetables than meat | 1 |
| balanced diet | 2 |
| more meat than vegetables | 3 |
| meat only | 4 |
| physical-exercise frequency |  |
| never do exercise | 0 |
| do exercise occasionally | 1 |
| more than once a week | 2 |
| every day | 3 |
| smoking addiction |  |
| never smoke | 0 |
| used to smoke, not smoke now | 1 |
| smoke now, but not every day | 2 |
| smoke everyday | 3 |
| alcohol addiction |  |
| never drink | 0 |
| drink occasionally | 1 |
| always drink but not every day | 2 |
| drink everyday | 3 |

a. The hypertension level is categorized according to the standard from American Heart Association.

No hypertension: DP < 80 mmHg, and SP < 120 mmHg

Elevated: DP < 80 mmHg, and 120 ≤ SP < 130 mmHg

high blood pressure stage 1: 80 ≤ DP < 89 mmHg, or 130 ≤ SP < 140 mmHg

high blood pressure stage 2: 90 ≤ DP < 120 mmHg, or 140 ≤ SP < 180 mmHg

high blood pressure crisis: DP ≥ 120 mmHg, or SP ≥ 180 mmHg
