# Supplementary material for: Protective and risk factors in daily life associated with cognitive decline of older adults
Source: Front Aging Neurosci. 2025 Feb 26;17:1496677. doi: 10.3389/fnagi.2025.1496677 (PMC11897038; doi:10.3389/fnagi.2025.1496677)
Supplement: Supplementary file 3 [file Table_3.DOCX]

**Raw codes**

S1 Codes of PCA

library(Factoshiny)

data(data.csv)

res.shiny <- PCAshiny(data.csv)

S2 Codes of Logistic regression

rm(list = ls())

library(readr)

test <- read_csv("test_lrm_68.csv")

train <- read_csv("train_lrm_68.csv")

data <- read_csv("E:/data_1/data.csv")

library(Formula)

library(ggplot2)

library(pROC)

library(rms)

ddDD=datadist(train)

options(datadist="ddDD")

formula <- as.formula(cognitive_deficit~gender + age + Hypertension_level + body_temperature + pulse_rate + self_assessment_of_health_condition + respiratory_rate + height + weight + BMI + dietary_habbit + physical_exercise_frequency + daily_physical_exercise_duration + physical_exercise_years + smoking_level + daily_Smoking + smoking_years + drinking_level + drinkin_years)

lrm <- lrm(formula,

data = train,

x=TRUE,

y=TRUE,

penalty=0,

tol=1e-15,

maxit=1000)

lrm

summary(lrm)

coef(lrm)

exp(coef(lrm))#odd.radio

train_pred_lrm <- predict(lrm,

newdata=train,

type = "fitted")

S3 ROC plotting

ROC_train_lrm <- roc(train$cognitive_deficit,train_pred_lrm,ci=TRUE)

ROC_train_lrm

plot(1-ROC_train_lrm$specificities,

ROC_train_lrm$sensitivities,type = "l",

col="red",

lty=1,

xlab = "1-Specificity",

ylab = "Sensitivity",

lwd=2,main='logistic lrm—train'

)

abline(0,1)

legend(0.25,0.233,

c("Area under the curve: 0.683

95% CI: 0.6561-0.71 (DeLong)"),

lty = c(1),

lwd = c(2),

col = c("red"),

bty = "2")

test_pred_lrm <- predict(lrm,

newdata=test,

type="fitted")

ROC_test_lrm <- roc(test$cognitive_deficit,test_pred_lrm,ci=TRUE)

ROC_test_lrm

plot(1-ROC_test_lrm$specificities,

ROC_test_lrm$sensitivities,type = "l",

col="red",

lty=1,

xlab = "1-Specificity",

ylab = "Sensitivity",

lwd=2,main='logistic lrm-test')

abline(0,1)

legend(0.255,0.233,

c("Area under the curve: 0.682

95% CI: 0.6399-0.7241 (DeLong)"),

lty = c(1),

lwd = c(2),

col = c("red"),

bty = "0")

S4 Nomograph plotting

library(survival)

library(lattice)

library(ggplot2)

library(rms)

daDa=datadist(data)

options(datadist="daDa")

fit <- lrm(formula,data = data,x=T,y=T)

fit

nom<- nomogram(fit, fun=plogis,

fun.at=c(0.0001,0.1,0.2,0.3,0.4,0.5,0.6,0.7,0.8,0.9,0.9999),

lp=F,

funlabel="cognitive_deficit")

plot(nom)

library(regplot)

nom5<-regplot(fit,

observation=data[1,],

center=TRUE,

title="Nomogram",

point=TRUE,

odds=TRUE,

showP=TRUE,

rank="sd",

clickable = FALSE)

regplot(fit,

observation=data[1,],

odds=TRUE,

interval="confidence",

points = TRUE)

S5 Calibration curve plotting

library(rmda)

cal1 <- calibrate(fit, method='boot', B=1000)

plot(cal1,xlim=c(0,1.0),ylim=c(0,1.0))
