# Supplementary material for: Protective and risk factors in daily life associated with cognitive decline of older adults
Source: Front Aging Neurosci. 2025 Feb 26;17:1496677. doi: 10.3389/fnagi.2025.1496677 (PMC11897038; doi:10.3389/fnagi.2025.1496677)
Supplement: Supplementary file 4 [file Table_4.DOCX]

S1 Principal components of Dimension 1 in figure 1B

| **Characteristics** | **Correlation** | **P Value** |
| --- | --- | --- |
| smoking years | 0.8274 | 0.000E+00 |
| smoking addiction | 0.8032 | 0.000E+00 |
| daily-cigarette number | 0.7473 | 0.000E+00 |
| alcohol addiction | 0.6394 | 0.000E+00 |
| drinking years | 0.6334 | 0.000E+00 |
| gender | 0.6200 | 0.000E+00 |
| height | 0.5619 | 3.313E-307 |
| weight | 0.4691 | 5.755E-202 |
| diastolic pressure | 0.1294 | 2.641E-15 |
| physical-exercise frequency | 0.1020 | 4.181E-10 |
| BMI | 0.08673 | 1.254E-07 |
| daily-physical-exercise duration | 0.08275 | 4.611E-07 |
| physical-exercise years | 0.07133 | 1.398E-05 |
| systolic pressure | 0.0575 | 4.646E-04 |
| hypertension level | 0.05416 | 9.775E-04 |
| age | -0.1415E | 5.003E-18 |

R^2^ = 0.01516 P = 5.616E-14

S2 Principal components of Dimension 2 in figure 1B

| **Characteristics** | **Correlation** | **P Value** |
| --- | --- | --- |
| physical-exercise frequency | 0.8219 | 0.000E+00 |
| Physical-exercise years | 0.7896 | 0.000E+00 |
| daily-physical-exercise duration | 0.7369 | 0.000E+00 |
| weight | 0.1550 | 2.452E-21 |
| systolic pressure | 0.1539 | 4.679E-21 |
| hypertension level | 0.1380 | 3.314E-17 |
| pulse rate | 0.1204 | 2.018E-13 |
| height | 0.1163 | 1.255E-12 |
| gender | 0.09239 | 1.784E-08 |
| diastolic pressure | 0.09195 | 2.088E-08 |
| BMI | 0.07271 | 9.500E-06 |
| age | -0.06713 | 4.351E-05 |
| body temperature | -0.1462 | 3.984E-19 |
| smoking addiction | -0.1783 | 8.155E-28 |
| daily-cigarette number | -0.1837 | 1.898E-29 |
| smoking years | -0.1868 | 1.963E-30 |

R^2^ = 0.001692 P = 1.230E-2
