# Supplementary material for: Protective and risk factors in daily life associated with cognitive decline of older adults
Source: Front Aging Neurosci. 2025 Feb 26;17:1496677. doi: 10.3389/fnagi.2025.1496677 (PMC11897038; doi:10.3389/fnagi.2025.1496677)
Supplement: Supplementary file 5 [file Table_5.DOCX]

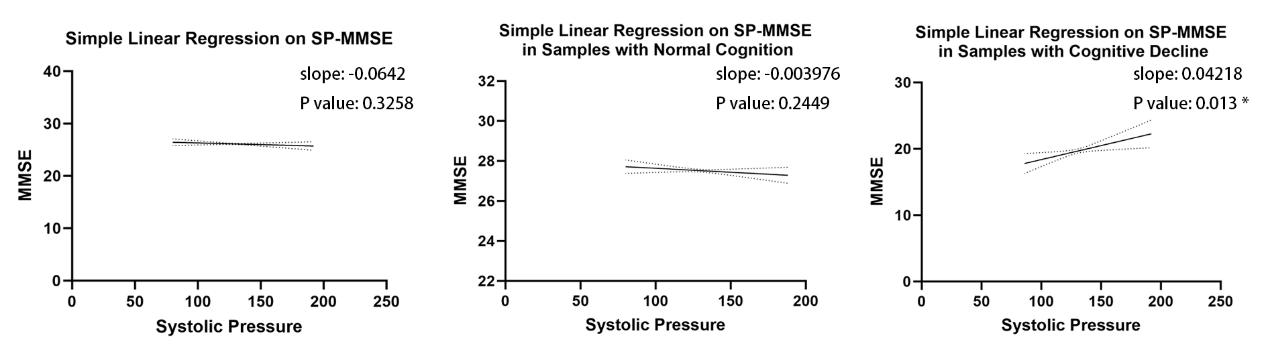


Simple linear regression between MMSE and systolic pressure.

The MMSE score was negatively correlated with systolic pressure in all the samples without significance (P = 0.3258). In the group of normal cognition, MMSE score and systolic pressure had a non-significant negative relationship (P = 0.2449). As for the older adults with cognitive decline, the correlation between MMSE and systolic pressure was positive with significance (P = 0.013).
